# Thoracic SMARCA4-deficient undifferentiated tumor: diagnostic characteristics, molecular insights, and treatment approaches

**DOI:** 10.1093/oncolo/oyaf121

**Published:** 2025-05-27

**Authors:** Junmin Song, Adam Gersten, Benjamin Herzberg, Balazs Halmos

**Affiliations:** Department of Medicine, Jacobi Medical Center, Albert Einstein College of Medicine, Bronx, NY 10461, United States; Department of Pathology, Montefiore Medical Center, Albert Einstein College of Medicine, Bronx, NY 10467, United States; Department of Hematology/Oncology, Columbia University, New York, NY 10032, United States; Department of Medical Oncology, Montefiore Medical Center, Albert Einstein College of Medicine, Bronx, NY 10461, United States

**Keywords:** SMARCA4-UT, SMARCA4-DTS, SMARCA4, BRG1, mediastinal tumor

## Abstract

Thoracic SMARCA4-deficient undifferentiated tumor (SMARCA4-UT) is a newly defined disease entity characterized by aggressive clinical behavior, diagnostic complexity, and poor prognosis. Given its rarity and heterogeneous histological presentation, establishing a definitive diagnosis can be challenging and relies heavily on comprehensive molecular and immunohistochemical evaluation. Here, illustrating the challenges highlighted by a real-world case, we describe the diagnostic pathway leading to SMARCA4-UT identification and discuss the differential diagnosis of thoracic tumors, relevant molecular evaluations, the molecular basis of SMARCA4-UT, and treatment implications. Recognition of this recently described entity is crucial, as an accurate diagnosis—as demonstrated by our case—can guide effective therapy and facilitate enrollment in targeted clinical trials.

**Key points**

- SMARCA4-UT is a recently recognized aggressive malignancy characterized by loss or severe reduction of SMARCA4 expression.
- Comprehensive immunohistochemical and molecular testing can differentiate SMARCA4-UT from other key tumor types.
- Integration of immune checkpoint inhibitors into treatment may yield benefits while several novel treatment options are currently under investigation.

## Patient story

**Figure 1.**
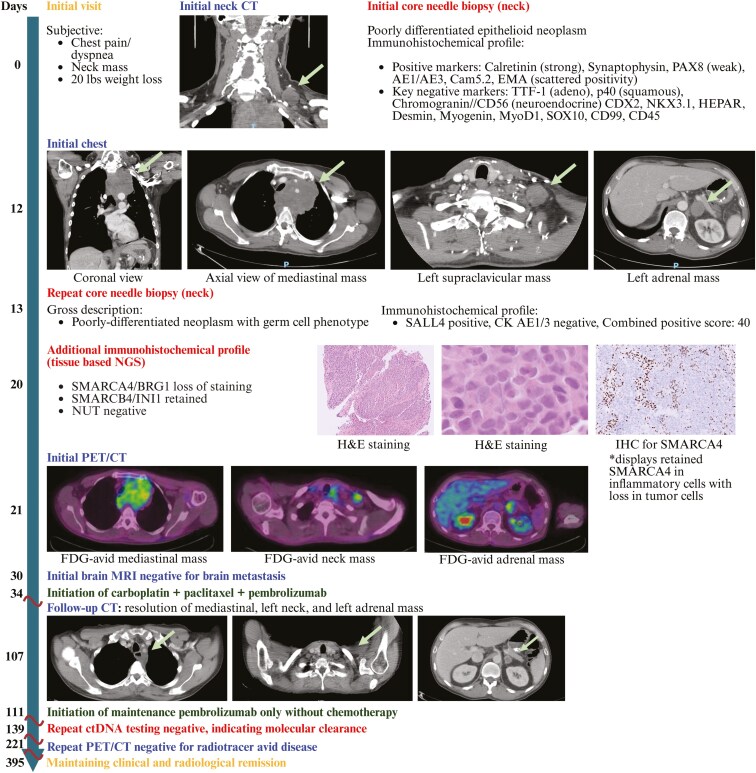
Visual summary of the patient’s clinical course. The patient’s initial presentation, diagnostic timeline, imaging findings (CT, PET/CT, MRI), biopsy results, immunohistochemical profiles, and treatment administered are described.

A 54-year-old Hispanic male with an over 30-year smoking history presented to the clinic with a growing left neck mass. He had excellent baseline performance status but experienced significant weight loss, episodic chest pain, and neck pain in the weeks before presentation. An initial core needle biopsy of the neck mass showed a poorly differentiated epithelioid malignant neoplasm. Immunohistochemical (IHC) staining was non-specific for tumor origin, showing positivity for calretinin, weak positivity for synaptophysin and PAX8, and scattered staining for AE1/3, Cam5.2, and EMA. Negative markers included CK7, CK20, chromogranin, CD56, p40, inhibin, TTF-1, CDX2, NKX3.1, HEPAR, desmin, myogenin, MyoD1, SOX10, CD99, and CD45 (Figure 1).

Neck CT revealed a 2.9 cm × 2.9 cm necrotic enhancing lesion in the left supraclavicular region and a mass in the superior mediastinum. Subsequent chest CT showed a bulky 9.8 cm heterogeneous anterior mediastinal mass extending from the aortic arch to the thyroid, displacing the trachea, along with a 4.5 cm necrotic left adrenal mass. Due to insufficient prior tissue, a repeat core needle biopsy of the neck mass was pursued and suggested a poorly differentiated neoplasm with a germ cell phenotype, as IHC staining was SALL4-positive but CK AE1/3-negative. PD-L1 combined positive score was 40. Serum AFP and beta-hCG were normal, but LDH was highly elevated. Testicular exam showed no mass. FDG PET-CT revealed a heterogeneously avid mediastinal mass, an FDG-avid left supraclavicular lymph node, and a left adrenal mass. Brain MRI was negative for metastasis.

Due to diagnostic challenges—germ cell phenotype, SALL4 positivity, weak PAX8 positivity suggesting mediastinal germ cell carcinoma, while clinical features argued for a poorly differentiated epithelial thoracic malignancy—the case was presented to the tumor board for further evaluation. Additional IHC was requested, demonstrating loss of SMARCA4/BRG1, retained SMARCB1/INI1, and negative staining for NUT. Expanded tissue-based next-generation sequencing (NGS) demonstrated SMARCA4/BRG1 loss, confirming a diagnosis of thoracic SMARCA4-deficient undifferentiated tumor (SMARCA4-UT). Circulating tumor DNA (ctDNA) testing detected a TP53 R249M mutation (37.7% of cell-free DNA) and CCND2 amplification.

## Molecular tumor board

The differential diagnosis for a mediastinal mass depends on the anatomical compartment involved—anterior/middle/posterior.^1^ Anterior compartment masses typically include thymic neoplasms, germ cell tumors, or thyroid-related masses. Middle compartment masses involve lymphadenopathy (reactive/infectious/metastatic) or foregut cysts, while posterior compartment masses are predominantly neurogenic tumors. Given our patient’s presentation with a large infiltrative anterior mediastinal mass and necrotic neck metastasis, the differential initially included thymic carcinoma, germ cell tumors, lung malignancies, or other metastatic cancers.

Pathological findings can narrow the differentials. In this case, the initial biopsy showed poorly differentiated epithelioid cells with a non-specific IHC profile. Tumor cells were weakly positive for synaptophysin and PAX8 but negative for markers commonly used to distinguish thymic, germ cell, and metastatic carcinomas. A second specimen initially suggested germ cell malignancy based on morphology, but germ cell stains and tumor markers were negative.

Tumors arising in midline locations should raise suspicion for NUT-rearranged, SMARCB1-deficient, or SMARCA4-deficient tumors.^2^ These tumors, traditionally associated with squamous histology, can mimic poorly differentiated or dedifferentiated tumors, such as germ cell tumors with IHC and NGS necessary for differentiation. NUT-rearranged tumors show positive NUT protein IHC and rearrangements like BRD4-NUT. SMARCB1-deficient tumors demonstrate loss of SMARCB1/INI1 expression, while SMARCA4-UT shows SMARCA4/BRG1 loss with retained SMARCB1/INI1 expression. Germ cell tumors express markers such as SALL4, OCT4, and PLAP, often along with isochromosome 12p[i(12p)]. Poorly differentiated carcinomas of head/neck origin, which typically express cytokeratin markers, p40, p63, p16 (HPV-related), or EBER (EBV-related), also need to be considered.

**Figure 2.**
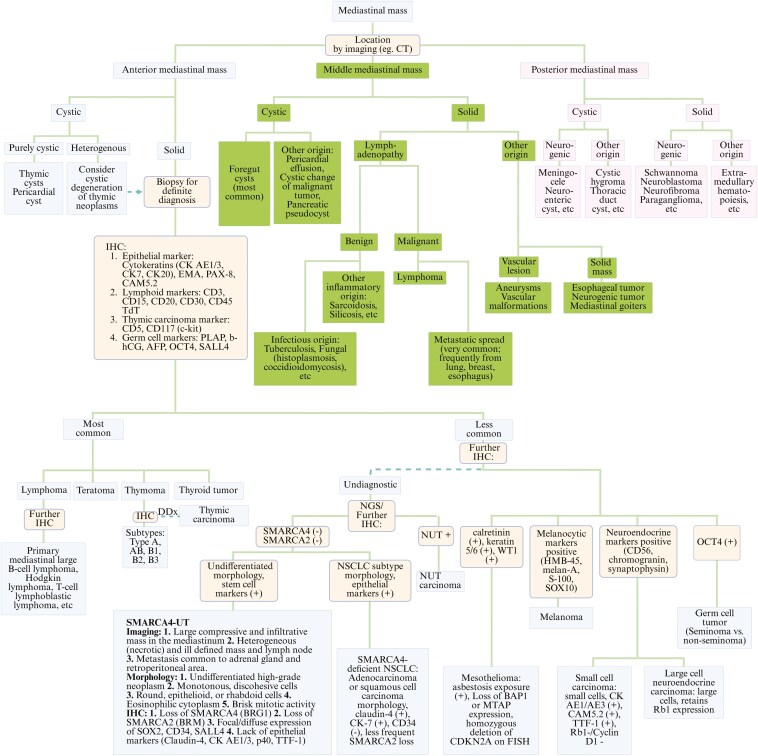
Differential diagnosis work-up for a mediastinal mass. A comprehensive diagnostic flowchart outlining the step-by-step evaluation and immunohistochemical profiling used to differentiate among potential etiologies of a mediastinal mass.

In our case, multidisciplinary discussion led to IHC and NGS testing, which confirmed SMARCA4/BRG1 loss, preserved SMARCB1/INI1 expression, and the absence of other pathognomonic markers, establishing the diagnosis of SMARCA4-UT (**Figure 2**).

### SMARCA4-UT—new entity

Thoracic SMARCA4-UT was added to the WHO Classification of Lung Tumors in 2021, characterized primarily by loss of the SMARCA4 gene.^3^ Initially reported in 2015 as SMARCA4-deficient thoracic sarcoma (SMARCA4-DTS), it was reclassified as SMARCA4-UT to reflect its lack of differentiation and epithelial features, moving it into the category of “other epithelial tumors.”^2^ SMARCA4-UT is a rare malignancy with a strong male predominance (90%) and association with smoking (>90%). Patients typically present at a median age of 58, slightly younger than those with non-small-cell lung cancer (NSCLC).^2^ Radiologically, SMARCA4-UT presents as a large (median size 120 mm), heterogeneous, ill-defined mass that is compressive and infiltrative, often involving nearby organs such as the lung parenchyma.^4^ It is frequently accompanied by necrotic lymphadenopathy in the mediastinal, internal thoracic, and cervical-subclavian regions.^2^ Metastasis outside the thorax is common, with the adrenal glands being the most frequent site, followed by bone and retroperitoneal metastases.^4^ These tumors also show strong FDG avidity on PET-CT.^4^ Our case exhibited these characteristic features, raising suspicion for SMARCA4-UT.

### Molecular work up

Biopsy with IHC staining and molecular testing is the gold standard for diagnosing thoracic SMARCA4-UT.^5^ According to WHO Classification 2021, diagnostic criteria include: (1) thoracic tumor in adults, (2) undifferentiated high-grade cells that are monotonous and discohesive, (3) round/epithelioid cells with prominent nucleoli and vesicular nuclei, and (4) loss or severe reduction of SMARCA4 expression.^3,6^ Additional diagnostic features include SMARCA2 loss and CD34, SALL4, and/or SOX2 expression.^2,6^ While SMARCA4-UT resembles SMARCB1/INI1-deficient neoplasms histologically, it retains SMARCB1 expression.^6^ SMARCA4-UT typically lacks claudin-4, an epithelial marker, and may show focal or absent expression of other epithelial markers such as cytokeratin AE1/AE3, EMA, p40, and TTF-1.^5^

Differentiating SMARCA4-UT from overlapping entities is crucial, particularly SMARCA4-deficient NSCLC (SD-NSCLC), as approximately 10% of NSCLCs exhibit SMARCA4 inactivation.^7^ Morphologically, NSCLCs are typically adenocarcinomas or squamous cell carcinomas, whereas SMARCA4-UT shows rhabdoid features with highly eosinophilic cytoplasm.^5^ On IHC, NSCLCs with SMARCA4 deficiency are usually claudin-4 positive (> 95%), CD34 negative, and less frequently exhibit SMARCA2 deficiency (30%) or focal SOX2/SALL4 expression (10%-15%).^2,5,7^ Though they have these distinguishing features, the biological relationship between SMARCA4-UT and SD-NSCLC remains a subject of debate. Recent evidence from Rekhtman et al,^8^ analyzing 22 SMARCA4-UTs and 45 SD-NSCLCs, suggests that SMARCA4-UT represents dedifferentiated carcinomas rather than primary sarcomas. This is supported by molecular and genomic data, including the presence of NSCLC components in composite tumors, shared oncogenic drivers (eg, KRAS, STK11, KEAP1), and smoking-associated mutational signatures, emphasizing a continuum of epithelial differentiation loss. This model has important implications for therapeutic development (discussed below) and also suggests that the observed phenotypic differences relate more to tumor evolution than to differing tumor origins.

Other differentials include neuroendocrine carcinoma, large cell lymphoma, germ cell tumors, thymic carcinoma, and NUT carcinoma, which can be ruled out by positive SMARCA4 staining. Melanoma, mesothelioma, small cell carcinoma, large cell carcinoma, malignant rhabdoid tumor, neuroendocrine carcinoma, and small cell carcinoma of the ovary, hypercalcemic type (SCCOHT), can also present with SMARCA4 deficiency. Positive melanocytic markers can exclude melanoma, while mesothelioma and SCCOHT are distinguished by cytokeratin and WT1 positivity.^2^ Small cell, large cell, and neuroendocrine carcinomas typically show claudin-4 positivity and lack SOX2, CD34, and SALL4 expression.^2^ Malignant rhabdoid tumors, which occur predominantly in young children, are characterized by SMARCB1 loss^2^ (**Table 1**).

**Table 1.** Immunohistochemical profiles of key differential diagnoses in undifferentiated thoracic tumors.

Marker	SMARCA4-UT	NUT carcinoma	SMARCB1-deficient tumor	Germ cell tumor
SMARCA4 (BRG1)	**Always deficient**	Retained	Retained	Retained
SMARCA2 (BRM)	Mostly deficient	Retained	Retained	Retained
SMARCB1 (INI1)	Mostly retained	Retained	**Always deficient**	Retained
NUT	Negative	**Strongly positive**	Negative	Negative
SALL4	Often positive	Negative or focally positive	Variable	**Positive (highly sensitive)**
OCT3/4	Negative	Negative	Negative	**Positive (seminoma, embryonal carcinoma)**
CD30	Negative	Negative or focally positive	Negative	**Positive (embryonal carcinoma)**
PLAP	Negative	Negative	Negative	**Positive (seminoma, embryonal carcinoma)**
CK AE1/AE3	Negative or focally positive	Positive	Positive (diffuse)	Positive
EMA	Negative or focally positive	Positive	Variable	Negative or focal
p40/p63	Negative	Often positive	Negative or non-specific	Negative
TTF-1	Negative	Negative	Negative	Negative
Synaptophysin / Chromogranin	Negative or focal	Negative	Negative	Negative

### SMARCA4-UT—molecular basis

**Figure 3.**
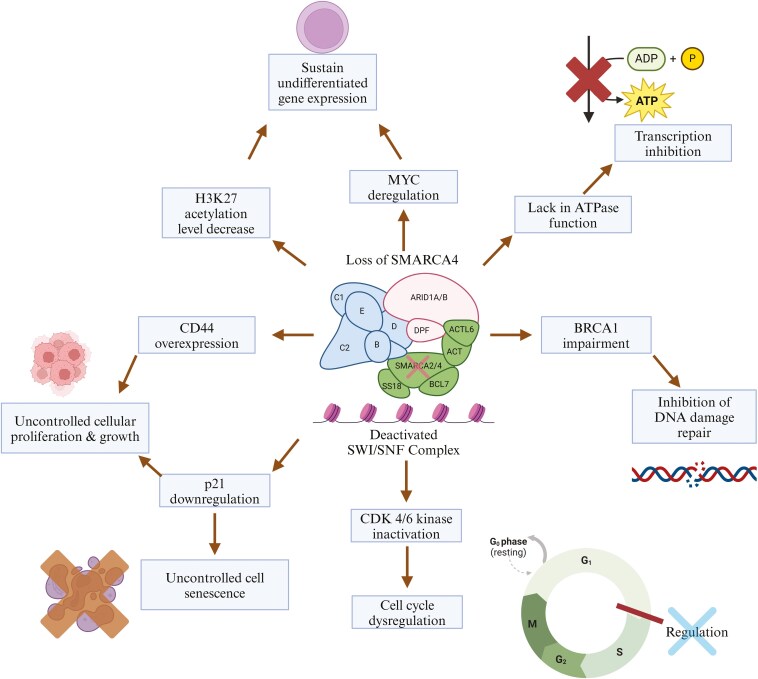
Molecular mechanisms of SMARCA4 loss and therapeutic vulnerabilities. Loss of SMARCA4 function leads to multiple dysregulated pathways, including MYC deregulation (promoting sustained undifferentiated gene expression), BRCA1 impairment (hindering DNA repair), and CDK4/6 kinase inactivation (leading to cell cycle dysregulation through disrupted pRb signaling). These alterations represent actionable therapeutic vulnerabilities, potentially targetable through therapies such as bromodomain and extra-terminal domain (BET) inhibitors (targeting MYC), poly (ADP-ribose) polymerase (PARP) inhibitors (targeting BRCA1-related DNA damage repair deficiencies), and CDK4/6 inhibitors (exploiting cell cycle dysregulation).

The SMARCA4 gene encodes BRG1, a crucial component of the switch/sucrose non-fermentable (SWI/SNF) chromatin-remodeling complex. SWI/SNF is a multi-subunit complex that regulates gene expression, DNA repair, and replication.^9^ Each complex contains an ATPase, either SMARCA4/BRG1 or SMARCA2/BRM, which provides energy for chromatin remodeling.^5^ Alterations in the SWI/SNF complex are found in over 20% of human cancers.^5^ SMARCA4 loss disrupts normal cellular functions, though the exact mechanisms remain unclear.^10^ It impacts tumorigenesis by downregulating cyclin D1, limiting cyclin-dependent kinase (CDK) 4/6 activity, and dysregulating the cell cycle.^5^ SMARCA4 loss also dephosphorylates pRb, downregulates p21, and promotes uncontrolled proliferation and senescence.^11^ Additionally, BRG1 regulates CD44 and interacts with BRCA1 to suppress tumors. SMARCA4 loss leads to CD44 overexpression and BRCA1 impairment, further driving proliferation and inhibiting DNA repair.^5,12^ Finally, BRG1 antagonizes MYC, and its loss allows cancer cells to activate growth factor receptors independently, preventing differentiation (**Figure 3**).^13^

### Treatment implications

As SMARCA4-UT is a newly identified entity, no standard treatment targeting its molecular alterations has been established.^5^ It generally carries a poor prognosis, with a median overall survival of 4.8 months.^14^ Surgery and radiation have limited benefits, with high recurrence rates even after negative margin resections. Chemotherapy, particularly paclitaxel plus carboplatin, is commonly used but has limited efficacy.^5^ Immune checkpoint inhibitors (ICIs) have shown promise due to the similarity between SMARCA4-UT and SMARCA4-deficient NSCLC.^14^ However, studies on ICIs have yielded mixed results, and treatment response does not consistently correlate with biomarkers such as PD-L1 expression, tumor mutation burden, or microsatellite stability status. These findings, however, are constrained by small sample sizes, highlighting the need for additional biomarkers such as cytokine signatures to better predict responses.^2,15,16^ A recent case series highlighted the possible importance of an immune-rich tumor microenvironment, particularly the presence of tertiary lymphoid structures, for ICI response in SMARCA4-UT, underscoring the need to identify further prognostic biomarkers.^17^ Additionally, a retrospective study demonstrated superior progression-free survival (mPFS 26.8 months vs. 2.73 months, *P* = .044) with combined ICI and chemotherapy compared to chemotherapy alone, suggesting the benefit of such a combined strategy.^14^ Also, the addition of anlotinib, a multitarget tyrosine kinase inhibitor, to ICI and chemotherapy has shown potential efficacy^14^ (Table 2).

**Table 2.** Current clinical trials applicable to SMARCA4-UT.

Drug	Trial number	Phase	Drug mechanism	Study population	Study size	Study result
Tazemetostat	NCT02601950	II	Selective EZH2 inhibition	Adults with INI1-negative solid tumors or synovial sarcoma	62	ORR: 15%, median PFS: 5.5 months
LY4050784	NCT06561685	I	Selective SMARCA2 (BRM) ATPase inhibition	Adults with locally advanced or metastatic solid tumor malignancy with SMARCA4 (BRG1) alteration	160 (estimate)	Currently enrolling
PRT3789	NCT05639751	I	SMARCA2 protein degrader	Adults with advanced, recurrent, or metastatic solid tumor malignancy with any SMARCA4 mutation or loss of function	226 (estimate)	Currently enrolling
PRT3789 + pembrolizumab	NCT06682806	II	SMARCA2 protein degrader + PD-1 inhibition	Adults with advanced, recurrent, or metastatic solid tumor malignancy with any SMARCA4 mutation or loss of function	60 (estimate)	Currently enrolling
PRT7732	NCT06560645	I	SMARCA2 protein degrader	Adults with advanced, recurrent, or metastatic solid tumor malignancy with any mutation of SMARCA4 by local testing that has either progressed on or is ineligible for standard of care therapy	104 (estimate)	Currently enrolling
FHD-286-C-002	NCT04891757	I	SMARCA4/SMARCA2 dual inhibitor	16 years or older patients with relapsed/refractory AML, MDS, or CMML	144 (estimate)	Currently enrolling
Tazemetostat + nivolumab + ipilimumab	NCT05407441	I/II	Selective EZH2 inhibition + PD-1 & CTLA-4 inhibition	Children with INI1- or SMARCA4-deficient tumors	49 (estimate)	Currently enrolling
Tiragolumab +- atezolizumab	NCT05286801	I/II	TIGIT inhibition +- PD-L1 inhibition	Children over 12 months of age with SMARCB1 or SMARCA4 deficient tumor	86 (estimate)	Currently enrolling
Nivolumab + ipilimumab	NCT04416568	II	PD-1 & CTLA-4 inhibition	Children aged 6 months to 30 years with relapsed or refractory INI1-negative cancers	45 (estimate)	Currently enrolling

Experimental therapies targeting SMARCA4 deficiency are under investigation. As SMARCA4 is an epigenetic regulator of gene function, the most common therapeutic hypotheses have involved epigenetic modulation. Enhancer of zeste homolog 2 (EZH2) inhibitors, such as tazemetostat, have shown limited activity in SMARCA4-deficient solid tumors.^18^ Synthetic-lethal interactions between SMARCA4 loss and SMARCA2 inhibition have spurred the development of SMARCA2-directed therapies, including the SMARCA2 protein degrader PRT-3789 and SMARCA2/BRM-specific inhibitors (NCT06561685).^19,20^ Notably, however, most SMARCA4-UT cases exhibit loss or markedly reduced expression of SMARCA2, though IHC may fail to detect low-level expression accurately.^21^ This raises the question of whether anti-SMARCA2 strategies will truly be effective for SMARCA4-UT, or only for SMARCA4-deficient NSCLC. It is also possible that epigenetic system targeting alone is insufficient to generate responses in most patients, and so emerging immuno-oncology strategies, such as combining EZH2 inhibitors or SMARCA4 protein degraders with ICIs, represent a promising area of exploration. Epigenetic modulation through these combinations could potentially reverse immune-cold tumor phenotypes, further enhancing treatment efficacy in SMARCA4-UT.^22-24^ Lastly, preclinical strategies, such as CDK4/6 inhibitors and ataxia telangiectasia-mutated and rad3-related (ATR) kinase inhibitors, are also being explored.^25,26^

## Patient update

The patient began chemo/immunotherapy with carboplatin, paclitaxel, and pembrolizumab, tolerating it well. The left neck mass rapidly shrank during the first cycle. After 4 cycles, follow-up CT showed significant improvement: resolution of left supraclavicular lymphadenopathy and tracheal narrowing, with a marked decrease in the left adrenal mass. Repeat ctDNA testing showed clearance, with the previously detected *TP53* mutation no longer detectable. He transitioned to maintenance pembrolizumab, administered every six weeks, and has maintained clinical and radiographic remission for over a year. Treatment benefits yielded improved appetite and energy, weight gain, and improved left shoulder function. With his improved physical condition and well-being, he has returned to full-time work and is able to care for an aging family member.

## Data Availability

Deidentified patient data and data relevant to the discussion in this manuscript are available from the corresponding author upon reasonable request.

## References

[1] Duwe BV , Sterman DH, Musani AI. Tumors of the mediastinum. Chest. 2005;128:2893-2909. https://doi.org/10.1378/chest.128.4.2893 16236967

[2] Nambirajan A , Jain D. Recent updates in thoracic SMARCA4-deficient undifferentiated tumor. Semin Diagn Pathol. 2021;38:83-89. https://doi.org/10.1053/j.semdp.2021.06.001 34147303

[3] Nicholson AG , Tsao MS, Beasley MB, et al The 2021 WHO Classification of Lung Tumors: Impact of Advances Since 2015. J Thorac Oncol. 2022;17:362-387. https://doi.org/10.1016/j.jtho.2021.11.003 34808341

[4] Crombé A , Alberti N, Villard N, et al Imaging features of SMARCA4-deficient thoracic sarcomas: a multi-centric study of 21 patients. Eur Radiol. 2019;29:4730-4741. https://doi.org/10.1007/s00330-019-06017-x 30762113

[5] Jiang J , Chen Z, Gong J, Han N, Lu H. Thoracic SMARCA4-deficient undifferentiated tumor. Discov Oncol. 2023;14:51. https://doi.org/10.1007/s12672-023-00639-w 37115343 PMC10147882

[6] Shinno Y , Ohe Y; Lung Cancer Study Group of the Japan Clinical Oncology Group (JCOG). Thoracic SMARCA4-deficient undifferentiated tumor: current knowledge and future perspectives. Jpn J Clin Oncol. 2024;54:265-270. https://doi.org/10.1093/jjco/hyad175 38117955

[7] Alessi JV , Ricciuti B, Spurr LF, et al SMARCA4 and other SWItch/sucrose nonfermentable family genomic alterations in NSCLC: clinicopathologic characteristics and outcomes to immune checkpoint inhibition. J Thorac Oncol. 2021;16:1176-1187. https://doi.org/10.1016/j.jtho.2021.03.024 33845210

[8] Rekhtman N , Montecalvo J, Chang JC, et al SMARCA4-deficient thoracic sarcomatoid tumors represent primarily smoking-related undifferentiated carcinomas rather than primary thoracic sarcomas. J Thorac Oncol. 2020;15:231-247. https://doi.org/10.1016/j.jtho.2019.10.023 31751681 PMC7556987

[9] Michel BC , D’Avino AR, Cassel SH, et al A non-canonical SWI/SNF complex is a synthetic lethal target in cancers driven by BAF complex perturbation. Nat Cell Biol. 2018;20:1410-1420. https://doi.org/10.1038/s41556-018-0221-1 30397315 PMC6698386

[10] Fernando TM , Piskol R, Bainer R, et al Functional characterization of SMARCA4 variants identified by targeted exome-sequencing of 131,668 cancer patients. Nat Commun. 2020;11:5551. https://doi.org/10.1038/s41467-020-19402-8 33144586 PMC7609548

[11] Kang H , Cui K, Zhao K. BRG1 controls the activity of the retinoblastoma protein via regulation of p21CIP1/WAF1/SDI. Mol Cell Biol. 2004;24:1188-1199. https://doi.org/10.1128/MCB.24.3.1188-1199.2004 14729964 PMC321457

[12] Bochar DA , Wang L, Beniya H, et al BRCA1 is associated with a human SWI/SNF-related complex: linking chromatin remodeling to breast cancer. Cell. 2000;102:257-265. https://doi.org/10.1016/s0092-8674(00)00030-1 10943845

[13] Romero OA , Setien F, John S, et al The tumour suppressor and chromatin-remodelling factor BRG1 antagonizes Myc activity and promotes cell differentiation in human cancer. EMBO Mol Med. 2012;4:603-616. https://doi.org/10.1002/emmm.201200236 22407764 PMC3407948

[14] Lin Y , Yu B, Sun H, et al Promising efficacy of immune checkpoint inhibitor plus chemotherapy for thoracic SMARCA4-deficient undifferentiated tumor. J Cancer Res Clin Oncol. 2023;149:8663-8671. https://doi.org/10.1007/s00432-023-04806-y 37115272 PMC10374696

[15] Chen J , Zheng Q, Wang J, Zhang X, Lv Y. Efficacy of immune checkpoint inhibitors in SMARCA4-deficient and TP53 mutant undifferentiated lung cancer. Medicine (Baltim). 2024;103:e36959. https://doi.org/10.1097/MD.0000000000036959 PMC11309689 38394494

[16] Shinno Y , Yoshida A, Masuda K, et al Efficacy of immune checkpoint inhibitors in SMARCA4-deficient thoracic tumor. Clin Lung Cancer. 2022;23:386-392. https://doi.org/10.1016/j.cllc.2022.03.005 35618627

[17] Gantzer J , Davidson G, Vokshi B, et al Immune-desert tumor microenvironment in thoracic SMARCA4-deficient undifferentiated tumors with limited efficacy of immune checkpoint inhibitors. Oncologist. 2022;27:501-511. https://doi.org/10.1093/oncolo/oyac040 35278076 PMC9177113

[18] Jones RL , Blay JY, Agulnik M, et al A phase II, multicenter study of the EZH2 inhibitor tazemetostat in adults (rhabdoid tumor cohort) (NCT02601950). Ann Oncol. 2018;29:viii580-viii581. https://doi.org/10.1093/annonc/mdy299.011

[19] Guo R , Dowlati A, Dagogo-Jack I, et al 603O First clinical results from a phase I trial of PRT3789: a first-in-class intravenous SMARCA2 degrader, in patients with advanced solid tumors with a SMARCA4 mutation. Ann Oncol. 2024;35:S483-S484. https://doi.org/10.1016/j.annonc.2024.08.670

[20] DiNardo CD , Savona MR, Kishtagari A, et al Preliminary results from a phase 1 dose escalation study of FHD-286, a novel BRG1/BRM (SMARCA4/SMARCA2) inhibitor, administered as an oral monotherapy in patients with advanced hematologic malignancies. Blood. 2023;142:4284-4284. https://doi.org/10.1182/blood-2023-178090

[21] Rossi G , Ragazzi M, Tamagnini I, et al Does immunohistochemistry represent a robust alternative technique in determining drugable predictive gene alterations in non-small cell lung cancer? Curr Drug Targets. 2017;18:13-26. https://doi.org/10.2174/1389450116666150330114441 25901525

[22] Shi Y , Shin DS. Dysregulation of SWI/SNF chromatin remodelers in NSCLC: its influence on cancer therapies including immunotherapy. Biomolecules. 2023;13:984. https://doi.org/10.3390/biom13060984 37371564 PMC10296130

[23] Herzberg B , Gandhi N, Henick BS, et al Effects of mutations in SWI/SNF subunits on context-specific prognosis in driver positive and driver negative NSCLC. J Clin Oncol. 2023;41:9039-9039. https://doi.org/10.1200/jco.2023.41.16_suppl.9039

[24] Chaudhri A , Lizee G, Hwu P, Rai K. Chromatin remodelers are regulators of the tumor immune microenvironment. Cancer Res. 2024;84:965-976. https://doi.org/10.1158/0008-5472.CAN-23-2244 38266066

[25] Kurashima K , Kashiwagi H, Shimomura I, et al SMARCA4 deficiency-associated heterochromatin induces intrinsic DNA replication stress and susceptibility to ATR inhibition in lung adenocarcinoma. NAR Cancer 2020;2:zcaa005. https://doi.org/10.1093/narcan/zcaa005 34316685 PMC8210217

[26] Xue Y , Meehan B, Macdonald E, et al CDK4/6 inhibitors target SMARCA4-determined cyclin D1 deficiency in hypercalcemic small cell carcinoma of the ovary. Nat Commun. 2019;10:558. https://doi.org/10.1038/s41467-018-06958-9 30718512 PMC6361890

